# Community Composition and Metabolic Potential of Endophytic Actinobacteria From Coastal Salt Marsh Plants in Jiangsu, China

**DOI:** 10.3389/fmicb.2019.01063

**Published:** 2019-05-14

**Authors:** Pan Chen, Chunmei Zhang, Xiuyun Ju, Youwei Xiong, Ke Xing, Sheng Qin

**Affiliations:** The Key Laboratory of Biotechnology for Medicinal Plant of Jiangsu Province, School of Life Sciences, Jiangsu Normal University, Xuzhou, China

**Keywords:** endophytic actinobacteria, coastal salt marsh, diversity, high throughput sequencing, metabolic potential, gene screening

## Abstract

The diversity and functional roles of the plant associated endophytic actinobacteria in unique habitats remain poorly understood. In this paper, we examined the phylogenetic diversity and community composition of endophytic actinobacteria associated with native coastal salt marsh plants in Jiangsu, China using a combination of cultivation and 16S rRNA gene-based high-throughput sequencing (HTS) methods. Further, we evaluated the antifungal, fibrinolytic activities and the secondary metabolite biosynthesis potential of isolates via gene screening. A total of 278 actinobacterial isolates were isolated from 19 plant samples. 16S rRNA gene sequencing revealed that the isolates were highly diverse and belonged to 23 genera within the *Actinomycetales* order, with *Streptomyces*, *Saccharopolyspora*, and *Pseudonocardia* comprising the most abundant genera. In addition, more than 10 of the isolates were novel actinobacterial taxa distributed across eight genera. HTS analyses of seven representative plant root samples revealed that Actinobacteria phylum constituted 0.04–28.66% of root endophytic bacterial communities. A total of four actinobacterial classes, 14 orders, 35 families, and 63 known genera were detected via HTS, and these communities were found to be dominated by the members of the order *Actinomycetales* including the genera *Streptomyces*, *Mycobacterium*, *Arthrobacter*, *Nocardioides*, and *Micromonospora*. In addition, 30.4% of the representative isolates exhibited antifungal activities, 40.5% of them showed fibrinolytic activities, while 43.0% of the strains harbored secondary metabolite biosynthesis genes. These results demonstrated that coastal salt marsh plants in the Jiangsu Province represented an underexplored new reservoir of diverse and novel endophytic actinobacteria that may be of potential interest in the discovery of bioactive compounds with potential as biocontrol agents and for fibrinolytic enzyme production.

## Introduction

The Actinobacteria phylum comprises Gram-positive and high guanine+cytosine (G+C) content bacterial taxa. Actinobacterial species are the main producers of active microbial natural products, are ubiquitously distributed among environments, and have been widely used in industrial and agricultural applications (Bèrdy, 2005; Qin et al., 2016). Recently, endophytic actinobacteria have particularly attracted significant attention, with increasing documentation of isolates from a wide range of plants, including various crop plants like wheat, rice, banana, apple, and tea plants, in addition to medicinal plants such as *Artemisia annua*, *Tripterygium wilfordii* Hook, *Glycyrrhiza inflata*, and *Jatropha curcas* (Conn and Franco, 2003; Tian et al., 2007; Qin et al., 2011, 2015b; Li et al., 2012; Miao et al., 2015; Álvarez-Pérez et al., 2017; Wei et al., 2018; Zhao et al., 2018). *Streptomyces*, *Micromonospora*, *Micrococcus*, *Pseudonocardia*, and *Microbacterium* are the most predominant endophytic actinobacterial genera cultivated, and many novel species from these genera have been identified in diverse host plants (Kaewkla and Franco, 2013; Golinska et al., 2015; Dinesh et al., 2017). Further, recent cultivation-independent analyses using 16S rRNA gene-based methods like denaturing gradient gel electrophoresis (DGGE), clone library analyses, and high-throughput sequencing have also revealed abundant endophytic actinobacterial communities within plants, including novel and uncultured taxa (Qin et al., 2012, 2018; Purushotham et al., 2018). For example, the high-throughput sequencing (HTS) of 16S rRNA genes from banana shoot tips yielded the detection of more than 50 known actinobacterial genera, which included many genera rarely identified as endophytes such as *Actinomycetospora*, *Actinoallomurus*, *Kibdelosporangium*, and *Kitasatospora* (Du et al., 2018).

In addition to the high species diversity of endophytic actinomycetes, increasing attention has been directed toward their ability to produce abundant bioactive metabolites. Several endophytic actinobacteria from medicinal plants produce novel bioactive compounds that have favorable antimicrobial and antitumor activities, besides showing a good potential for pharmaceutical development and biotechnological applications (Qin et al., 2011; Golinska et al., 2015; Dinesh et al., 2017). For example, the recently identified hamuramicins A and B obtained from the endophytic actinomycete *Allostreptomyces* sp. K12-0794 exhibited both antimicrobial activity and human cell line toxicity (Suga et al., 2018). In addition to their therapeutic uses, endophytic actinomycetes have a broad potential and value in agricultural application. The endophytes can promote plant growth via direct and indirect mechanisms, including phosphorus solubilization, production of 1-aminocyclopropane-1-carboxylic acid (ACC) deaminase, siderophores, induction of pathogen resistance, and aiding resistance to abiotic stresses like those imposed by high salinity and drought (Conn et al., 2008; Qin et al., 2014, 2017; Trujillo et al., 2015; Zeng et al., 2018).

When compared with the well-studied crop plants and endophytic actinobacteria within common environments, little is known about naturally occurring plant-associated actinobacteria within harsh habitats like hot and cold deserts, saline and acidic soils, and marine habitats. Coastal salt marshes are one of the most biologically productive habitats on Earth, and are one such example of a unique and harsh environment (Pennings and Bertness, 2001). Recent studies from coastal sediments and mangrove forests have revealed high levels of actinobacterial diversity (Hong et al., 2009; Hamedi et al., 2013; Jiang et al., 2018). Surprisingly, however, there is still little information related to the isolation, diversity and their biological activity of endophytic actinobacteria within coastal salt marsh ecosystems, despite the fact that such environments are likely to contain unique and phylogenetically diverse endophytes. Our recent analyses have indicated that there were abundant Actinobacteria and novel taxa in the rhizosphere soils associated with halophytes collected from coastal salt marshes within the Jiangsu Province of China (Wang et al., 2015; Bai et al., 2016; Ding et al., 2018). In particular, the phylum Actinobacteria comprised 2.8%–43.0% of rhizosphere bacterial communities, as evidenced by HTS on the Illumina MiSeq platform (Gong et al., 2018). Importantly, rhizosphere soils can be a source of endophytes (Long et al., 2010). There are abundant halophyte resources in the Jiangsu coastal region. Consequently, we speculated that plants within the Jiangsu coastal zone also contained abundant endophytic actinobacteria.

Thus, the aims of this study were to (i) survey the diversity of culturable endophytic actinobacteria from plants within coastal salt marshes in the Jiangsu province, (ii) analyze root endophytic actinobacterial communities from representative plants using 16S rRNA gene-based HTS technique, (iii) evaluate the anti-phytopathogenic fungal and fibrinolytic activity and analyze secondary metabolite biosynthetic gene profiles of isolates. We expect to provide a preliminary basis for the further discovery of biological active metabolites from coastal salt marsh plants endophytic actinobacteria and their further utilization for biocontrol of crops and thrombosis treatment for human beings.

## Materials and Methods

### Sample Collection

Plant samples were collected from the coastal salt marsh region of the Jiangsu Province in Eastern China (32°26′09–34°45′14 N, 119°14′16–121°19′30 S) between 2010 and 2014. Nearly 20 healthy plant samples were collected, including roots, stems, and leaf samples from herbs, grasses, and woody trees. Representative plants sampled in the study included *Tamarix chinensis* Lour., *Dendranthema indicum*, *Salicornia europaea* Linn., *Sesbania cannabina*, *Spartina alterniflora*, and *Suaeda glauca*. Samples were placed in sterile plastic bags and transported to the laboratory within 24 h, where they were either stored at 4°C for analysis within one to 3 days or at -80°C for DNA extraction that was conducted within 2 weeks.

### Isolation of Endophytic Actinobacterial Isolates

Plant surface sterilization and evaluation of sterilization effectiveness were conducted following our previously described five-step procedure (Qin et al., 2009). The sterilization time used for each sample varied based on the specific plant tissues and organ types. Surface-sterilized samples were then aseptically grinded into smaller fragments using a commercial blender and then spread onto seven different Actinobacteria-selective media types: (A) modified TWYE medium supplemented with 20% (v/v) plant extracts (Qin et al., 2009); (B) starch-casein agar (Xing et al., 2012); (C) sodium propionate agar (Qin et al., 2009); (D) ISP 5 agar (Shirling and Gottlieb, 1966); (E) trehalose-proline agar (Qin et al., 2009); (F) MOPS-amino acid agar [MOPS 2.0 g, (NH_4_)_2_SO_4_ 1.0 g, CaCl_2_ 2.0 g, K_2_HPO_4_ 0.5 g, amino acid mixture 1.0 g, agar 15.0 g, H_2_O 1000 mL, pH 7.2]; and (G) CMC agar (Kaewkla and Franco, 2013). All media were supplemented with 3% (w/v) NaCl, nystatin antibiotic (50 mg l^-1^), and nalidixic acid (50 mg l^-1^). Cultures were incubated at 28°C for 2 to 8 weeks. Colonies obtained after incubation were purified and then maintained on slants at 4°C and also as 30% (v/v) glycerol suspensions stored at -80°C.

### Isolates Identification

Isolates were preliminarily identified via morphologic and culture characteristics that were observed when growing on the International *Streptomyces* Project media ISP 2 and ISP 4 (Shirling and Gottlieb, 1966) that contained 3% NaCl. Diaminopimelic acid isomer analyses were used to distinguish *Streptomycetes* from other spore-forming actinomycetes following the methods described previously (Lechevalier and Lechevalier, 1980). The extraction of genomic DNA and PCR amplification of 16S rRNA genes for each representative isolate were conducted using the methods described previously (Li et al., 2007). The 16S rRNA gene was amplified using universal primer 27f (5′-CAGAGTTTGATCCTGGCT-3′) and 1492r (5′-AGGAGGTGATCCAGCCGCA-3′), and the similarities were determined by comparison of sequences against the EzTaxon-e database (Kim et al., 2012). Neighbor-joining phylogenetic trees of 16S rRNA gene sequences were constructed using the molecular evolutionary genetics analysis (MEGA) software program, version 6.0 (Tamura et al., 2013). A detailed identification of the potentially novel taxa was conducted using the conventionally used polyphasic taxonomic procedures (Stackebrandt et al., 2002).

### High Throughput Sequencing Analysis of Endophytic Actinobacteria

After surface sterilization, seven representative plant root samples of *Phragmites australis* (HR1), *Sesbania cannabina* (HR2), *Chrysanthemum indicum* (HR3), *Metaplexis japonica* (HR4), *Suaeda glauca* (HR5), *Lycium* Linn (HR6), and *Spartina alterniflora* (HR7) collected at the same time were subjected to total genomic DNA extraction by Shanghai Majorbio Biotechnology, Co., Ltd. (Shanghai, China). The V5-V7 hypervariable region of bacterial 16S ribosomal RNA genes were then amplified by PCR using an amplification procedure consisting of 95°C for 3 min, followed by 27 cycles at 95°C for 30 s, 55°C for 30 s, and 72°C for 45 s, and a final extension at 72°C for 10 min. PCRs incorporated the bacteria-specific primers 799F (5′-AACMGGATTAGATACCCKG-3′) (Chelius and Triplett, 2001) and 1115R (5′-AGGGTTGCGCTCGTTG-3′) (Reysenbach and Pace, 1995). PCR amplicons were extracted from 2% agarose gels and purified using the AxyPrep DNA Gel Extraction Kit (Axygen Biosciences, Union City, CA, United States) according to the manufacturer’s instructions, followed by quantification using the QuantiFluor^TM^ -ST system (Promega, United States). Purified amplicons were pooled in equimolar concentrations and paired-end sequenced (2 × 250) on the Illumina MiSeq platform at Majorbio Biotech, Co., Ltd. (Shanghai, China), according to standard protocols.

Raw fastq sequence files were demultiplexed and quality-filtered using QIIME version 1.17 (Caporaso et al., 2010). Operational taxonomic units (OTUs) were clustered at the 97% nucleotide similarity cutoff using the UPARSE software package version 7.1 (Edgar, 2013) while chimeric sequences were identified and removed using the UCHIME software package version 4.2.40 (Edgar et al., 2011). The phylogenetic affiliation of each 16S rRNA gene sequence was assessed using the RDP Classifier (Wang et al., 2007) and comparison against the SILVA 16S rRNA gene database while using a confidence threshold of 70% (Quast et al., 2013). Community alpha diversity values were evaluated using the mothur software version 1.30.1 (Schloss et al., 2009). The sequence data have been submitted to the NCBI Sequence Read Archive (SRA) database under the Accession No. PRJNA507498.

### Antifungal and Fibrinolytic Activity Assays of Endophytic Actinobacterial Isolates

Actinobacterial strains were screened *in vitro* for the growth inhibition of eight different fungi that were pathogenic to crops and trees: *Rhizoctonia solani*, *Pyricularia grisea*, *Verticillium dahlia* Kleb, *Marssonina brunnea* YH1, *Lasiodiplodia theobromae* YB3, *Sclerotium* sp. YF2, *Fusarium graminearum*, and *Botryosphaeria berengeriana*. All pathogens were maintained at the Key Laboratory of Biotechnology for Medicinal Plants within the Jiangsu province, China. The fermentation capacity of the isolates and their antimicrobial activities were determined using soybean mannitol liquid medium (Qin et al., 2009) containing 3% NaCl and the agar diffusion assay method (Romero et al., 2004). Briefly, each tested strain was cultured in soybean mannitol liquid medium (shaken at 160 rpm, 28°C) for 5 days. Then, the fermentation broth was used for antimicrobial activity screening. Ketoconazole was used as the positive control, and sterile water was used as the negative control. The fibrinolytic activity was determined by using the fibrin plate method according to Astrup and Müllertz (1952). Fibrinolytic activity was evaluated by measuring the diameter of the transparent zone. Urokinase was used as the positive control, and uninoculated medium was used as the negative control.

### Screening for Secondary Metabolite Genes

The potential for biosynthesis and production of secondary meta-bolites was evaluated for the isolates using PCR. The specific pri-mer pairs K1F (5′-TSAAGTCSAACATCGGBCA-3′)/M6R (5′-CGCAGGTTSCSGTACCAGTA-3′), KSα (5′-TSGCSTGCTTGGAYGCSATC-3′)/KSβ (5′-TGGAANCCG CCGAABCCTCT-3′), A3F (5′-GCSTACSYSATSTACACSTCSGG-3′)/A7R (5′-SASGTCVCCSGTSCGGTAS-3′), and Halo-B4-FW (5′-TTCCCSCGSTACCASATCGGSGAG-3′)/Halo-B7-RV (5′-GSGGGATSWMCCAGWACCASCC-3′) were used to determine the presence of polyketide synthase I (PKS I), polyketide synthase II (PKS II), non-ribosomal peptide synthetase (NRPS), and FADH_2_-dependent halogenase (Halo) genes, respectively (Metsä-Ketelä et al., 1999; Ayuso-Sacido and Genilloud, 2005; Hornung et al., 2007). PCRs were conducted following the methods described previously (Gao and Huang, 2009; Qin et al., 2009). Amplification products were determined by 1.0% agarose gel electrophoresis, and bands of size 1,200–1,400, 600, 700–800 and about 550 bp were considered positive PCR amplifications of PKS I, PKS II, NRPS, and Halo genes, respectively.

### GenBank Accession Numbers

16S rRNA gene sequences for the isolates obtained in this study were deposited at GenBank under the Accession Nos. JQ819252–JQ819262 and JX982699–JX982766.

## Results

### Isolation of Endophytic Actinobacteria

A total of 278 isolates were cultured from sampled plant tissues and the most abundant genus of isolates based on preliminary identifications was *Streptomyces* (∼60% of isolates). From the screening based on morphological criteria and cultural characteristics, 79 representative strains were selected for further investigation. The starch-casein and sodium propionate media cultures yielded the most isolates and the highest diversity of non-*Streptomyces* endophytic actinobacteria, with nine different genera cultivated including several unique rare genera. The ISP 5 medium yielded the next highest diversity of endophytic actinobacteria, while the other four media types (A, E, F, G) yielded only three to five genera each (Table 1). The majority of isolates were obtained from root samples (42 isolates, 53.2%), followed by leaves (20 isolates, 25.3%) and stems (17 isolates, 21.5%) (Table 2).

**Table 1:** The abundance and diversity of isolates belonging to different actinobacterial genera that were recovered with different media.

Isolation Medium	Total isolates	Different genera obtained using different medium	Number of genera
A	5	*Kocuria*, *Saccharopolyspora*, *Streptomyces*	3
B	23	*Amycolatopsis*, *Brevibacterium*, *Kocuria*, *Micrococcus, Micromonospora*, *Nocardiopsis, Pseudonocardia*, *Saccharopolyspora*, *Streptomyces*, *Tamaricihabitans*	10
C	19	*Glycomyces*, *Gordonia*, *Kocuria*, *Micrococcus*, *Modestobacter*, *Mycobacterium*, *Nocardiopsis*, *Pseudokineococcus*, *Saccharopolyspora*, *Streptomyces*	10
D	12	*Citricoccus*, *Janibacter*, *Kineococcus*, *Micrococcus*, *Prauserella*, *Pseudonocardia*, *Saccharopolyspora*, *Streptomyces*	8
E	6	*Nesterenkonia*, *Pseudonocardia*, *Rhodococcus*, *Saccharopolyspora*, *Streptomyces*	5
F	6	*Glycomyces*, *Janibacter*, *Nocardiopsis*, *Streptomyces*	4
G	8	*Dietzia*, *Glycomyces*, *Kocuria*, *Nocardia*, *Streptomyces*	5


*Medium: A, modified TWYE agar supplemented with plant extract (plant extracts were made from three different plants: *Tamarix chinensis* Lour., *Suaeda glauca*, and *Spartina alterniflora*); B, starch-casein agar; C, sodium propionate agar; D, ISP 5 agar; E, trehalose-proline medium; F, MOPS-amino acid agar; G, CMC agar.*

**Table 2:** Taxonomic distribution of endophytic actinobacterial isolates from different host plant tissues and their associated bioactivities, and the presence of secondary metabolite biosynthetic genes.

						No. of strains with antifungal activity	No. of strains with biosynthetic genes	No. of strains with fibrinodlytic activity
Taxonomic group		Sample tissues and number of isolates						
					
		Root	Stem	Leaf	Total			
Streptomycineae:					19
	Streptomyces	5	7	7	19	10	12	12
Pseudonocardineae:					25
	Pseudonocardia	5	1	2	8	7	2	2
	Saccharopolyspora	6	4	4	14	4	4	8
	Amycolatopsis		1		1		1	1
	Prauserella	1			1	1	
	Tamaricihabitans	1			1		1	1
Micromonosporineae:					1
	Micromonospora	1			1			1
Microococcineae:					15
	Brevibacterium			1	1	
	Janibacter	2			2	1	
	Kocuria	4		1	5		1	1
	Nesterenkonia	1			1		1	
	Micrococcus	1	2	2	5		2	
	Citriococcus			1	1		1	
Streptosporangineae:					3
	Nocardiopsis	2	1		3			3
Corynebacterineae:					9
	Gordonia	3			3		2	
	Dietzia	1			1	
	Mycobacterium	1			1		1	
	Nocardia	2			2			
	Rhodococcus	2			2	1	2	
Frankineae:					2
	Modestobacter	2			2		1	
Kineosporiineae:					2
	Kineococcus			1	1		1	
	Pseudokineococcus	1			1		1	
Glycomycineae:					3
	Glycomyces	1	1	1	3		1	3
Total		42	17	20	79	24	34	32


### Diversity of Isolated Endophytic Actinobacteria

**FIGURE 1:**
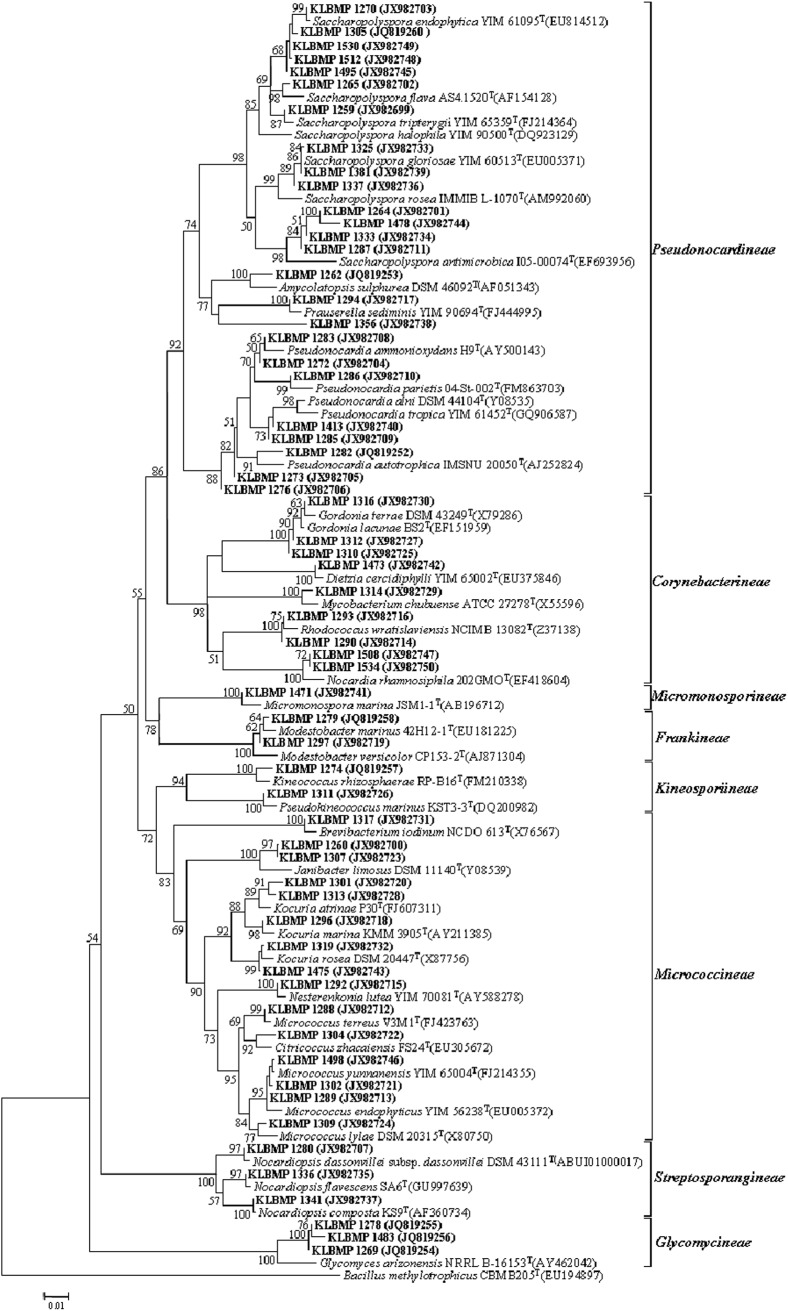
Neighbor-joining phylogenetic tree of 16S rRNA gene sequences obtained from non-*Streptomyces* isolates in this study and their phylogenetic neighbors. Only bootstrap values above 50% are shown at nodes and are expressed as percentages of 1,000 replicates. Bar represents 0.01 substitutions per nucleotide position. GenBank accession numbers of reference 16S rRNA gene sequences are shown next to isolate names. *Bacillus methylotrophicus* CBMB205^T^ (EU194897) was used as the outgroup.

**FIGURE 2:**
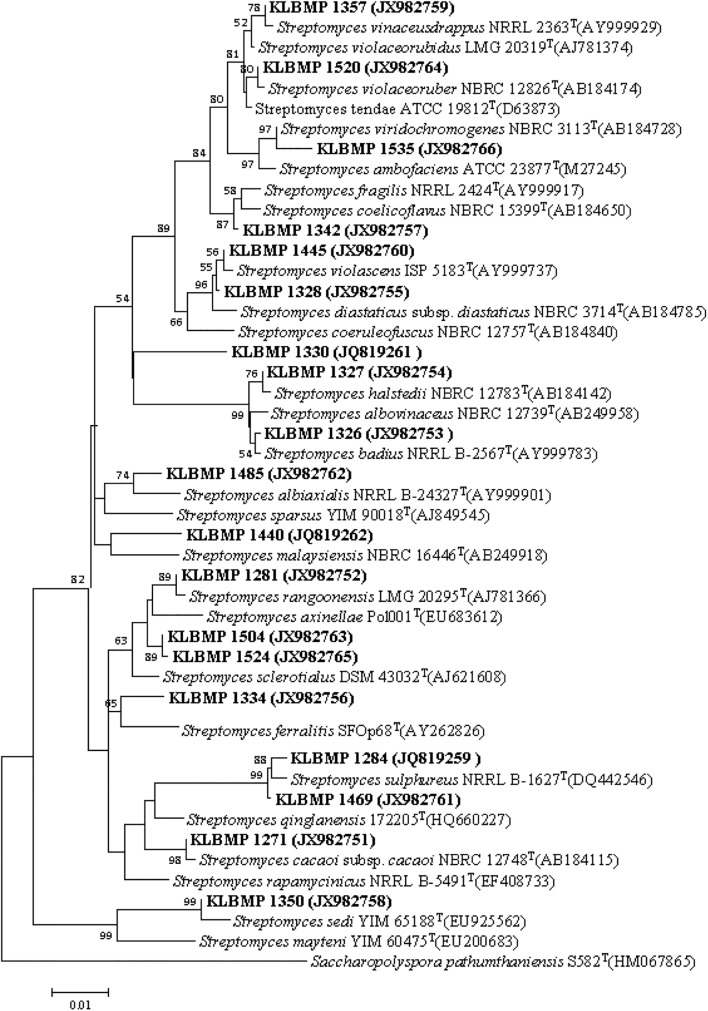
Neighbor-joining phylogenetic tree of 16S rRNA gene sequences obtained from *Streptomyces* isolates in this study and their phylogenetic neighbors. Only bootstrap values above 50% are shown at nodes and are expressed as percentages of 1,000 replicates. Bar represents 0.01 substitutions per nucleotide position. GenBank accession numbers of reference 16S rRNA gene sequences are shown next to isolate names. *Saccharopolyspora pathumthaniensis* S582^T^ (HM067865) was used as the outgroup.

16S rRNA gene sequence analysis of the 79 isolates revealed significant genetic diversity distributed among nine suborders within the *Actinobacteria* class: *Streptomycineae*, *Pseudonocardineae*, *Streptosporangineae*, *Micromonosporineae*, *Micrococcineae*, *Glycomycineae*, *Frankineae*, *Corynebacterineae*, and *Kineosporiineae*. The suborders comprised 14 families and 22 known genera in addition to the novel genus, *Tamaricihabitans* (Figure 1, 2 and Table 2). Except the *Streptomycineae* (19 isolates, one genus), rare actinobacterial strains of the family *Pseudonocardineae* (25 isolates, 5 genera), *Microococcineae* (15 isolates, six genera), and *Corynebacterineae* (9 isolates, 5 genera) were the most prominent among the isolates. *Streptomyces* was the most frequently isolated genus (24%, 19 isolates), followed by *Saccharopolyspora* (14 isolates) and *Pseudonocardia* (eight isolates). Fifteen genera including those rarely detected as endophytes such as *Dietzia*, *Kineococcus*, *Citriococcus*, and *Janibacter* were isolated less frequently and were each represented by only one or two strains. Genera *Prauserella*, *Modestobacter*, *Pseudokineococcus*, *Glycomyces*, and *Nesterenkonia* have only been rarely reported as culturable endophytes previously, and their presence in these samples underscores the high level of diversity within the plants of this environment. The root tissues yielded the majority of the genera (20), while stem and leaf tissues only yielded seven and nine genera, respectively (Table 2). The 16S rRNA gene sequence nucleotide identities ranged from 95.1 to 100% between strains and the available type strains. Interestingly, several strains were most closely related to other validly published endophytic actinobacteria from marine environments, but exhibited distinct phenotypic characteristics when compared with their marine relatives. For example, strain KLBMP 1279 shared 99.5% 16S rRNA gene sequence identity to *Modestobacter marinus* 42H12-1^T^, a marine obtained actinobacterium isolated from a deep-sea sediment sample (2,983 m depth) from the Atlantic Ocean (Xiao et al., 2011). However, KLBMP 1279 exhibited culture characteristics differing from *M. marinus* and thus represents a new *Modestobacter* species, named as *Modestobacter roseus* (Qin et al., 2013b). Moreover, several strains exhibited relatively lower 16S rRNA gene identities (<98.7%) to characterized isolates and represented novel species (Table 3). For example, two *Streptomyces* isolates, KLBMP 1440 and KLBMP 1330, shared only 98.5 and 98% 16S rRNA gene nucleotide similarities to their nearest characterized relatives, respectively. These values are lower than the suggested cutoff (98.7%) for species delineation, as suggested by Chun et al. (2018). Further, the 16S rRNA genes of these isolates also comprised distinct clades in the phylogenetic reconstructions (Supplementary Figures S1, S2). Consequently, the two strains were considered novel species of the genus *Streptomyces*. 16S rRNA gene sequence analysis led to the selection of 13 strains (16.5%) for detailed identification via standard polyphasic taxonomic identification methods, and eight of these strains (KLBMP 1356, KLBMP 1279, KLBMP 1305, KLBMP 1262, KLBMP 1269, KLBMP 1274, KLBMP 1282, and KLBMP 1284) were validated published as novel genus/species (Table 3). Overall, these results demonstrate the presence of abundant and diverse endophytic actinobacteria within plants that are native to coastal salt marshes.

**Table 3:** 16S rRNA gene sequence nucleotide similarities between novel endophytic isolates and the most closely related type strain of a validly described species.

Strains	Host plant	Tissues type	The closest phylogenetic type strains	16S rRNA gene sequence similarity (%)	References
KLBMP 1356 (Novel genus)	*Tamarix chinensis* Lour.	Root	*Prauserella marina* MS498^T^	95.1	Qin et al., 2015a
KLBMP 1279 (Novel species)	*Salicornia europaea* Linn.	Root	*Modestobacter marinus* 42H12-1^T^	99.5	Qin et al., 2013b
KLBMP 1305 (Novel species)	*Dendranthema indicum* (Linn.) Des Moul	Stem	*Saccharopolyspora pathumthaniensis* S582^T^	99.3	Zhang et al., 2013
KLBMP 1262 (Novel species)	*Dendranthema indicum* (Linn.) Des Moul	Stem	*Amycolatopsis sulphurea* DSM 46092^T^	97.9	Xing et al., 2013
KLBMP 1274 (Novel species)	*Limonium sinense* (Girard) Kuntze	Leave	*Kineococcus rhizosphaerae* RP-B16^T^	98.7	Bian et al., 2012
KLBMP 1282 (Novel species)	*Tamarix chinensis* Lour.	Leave	*Pseudonocardia kongjuensis* LM 157^T^	98.3	Xing et al., 2012
KLBMP 1284 (Novel species)	*Tamarix chinensis* Lour.	Stem	*Streptomyces sulphureus* NRRL B-1627^T^	99.4	Qin et al., 2013a
KLBMP 1469 (Novel species)	*Tamarix chinensis* Lour.	Leave	*Streptomyces sulphureus* NRRL B-1627^T^	99.5	
KLBMP 1330 (Novel species)	*Aster tataricus* L. F.	Root	*Streptomyces malachitospinus* NBRC 101004^T^	98.0	
KLBMP 1440 (Novel species)	*Tamarix chinensis* Lour.	Root	*Streptomyces yogyakartensis* NBRC 100779^T^	98.5	
KLBMP 1269 (Novel species)	*Limonium sinense* (Girard) Kuntze	Leave	*Glycomyces arizonensis* NRRL B-16153^T^	97.0	Xing et al., 2014
KLBMP 1483 (Novel species)	*Dendranthema indicum* (Linn.) Des Moul.	Stem	*Glycomyces arizonensis* NRRL B-16153^T^	96.7	
KLBMP 1278 (Novel species)	*Sesbania cannabina* (L.) Pers.	Root	*Glycomyces arizonensis* NRRL B-16153^T^	97.0	


### HTS of Endophytic Actinobacterial Diversity

**FIGURE 3:**
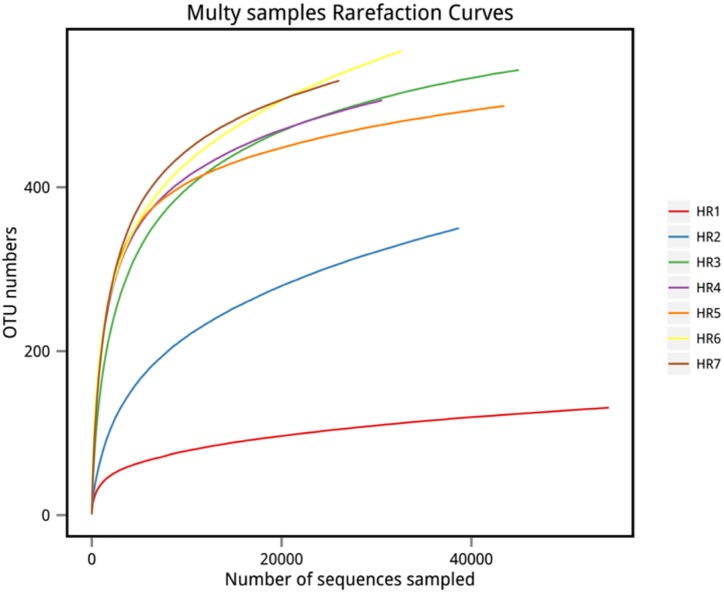
Rarefaction curves of OTU richness among samples. OTU richness was determined via sequencing of the V5–V7 hypervariable regions of 16S rRNA genes.

High-throughput sequencing on the Illumina MiSeq sequencing platform was conducted to explore the endophytic actinobacterial community within roots (where diversity was highest) of seven typical coastal plants. A total of 228,091 16S rRNA gene sequences were considered high quality, with 22,450–49,221 sequences obtained per sample. At the 97% nucleotide identity threshold for OTU identification, the number of OTUs ranged from 131 to 566 within samples (Table 4). An analysis of the coverage indicated that greater than 99% of the total diversity within the communities was sampled, while rarefaction analyses indicated that measured diversity was nearly saturated in most samples (Figure 3). Alpha diversity indices including the Chao, ACE, Shannon, and Simpson metrics were calculated and are provided in Table 4. These results indicated an abundant diversity of endophytic bacteria in the seven root samples.

**Table 4:** Alpha diversity estimates for bacterial communities within the seven plant root samples.

Sample ID	16S rRNA gene reads	OTUs (97%)	Coverage	Community richness		Community diversity	
					
				Chao^∗^	Ace	Simpson	Shannon
HR1	49,221	131	0.9993	183.000	184.5747	0.275	1.8406
HR2	35,125	350	0.9969	522.575	566.0036	0.312	2.0495
HR3	38,071	543	0.9982	587.652	601.6451	0.155	3.3998
HR4	26,919	506	0.9971	604.154	585.4462	0.024	4.5645
HR5	29,885	499	0.9985	555.447	563.5344	0.052	4.3357
HR6	26,420	566	0.9960	726.660	784.6813	0.032	4.5812
HR7	22,450	530	0.9966	617.000	593.0172	0.118	3.7663


*^∗^Chao and ACE indices reflect species richness, Shannon and Simpson indices reflect species diversity.*

**FIGURE 4:**
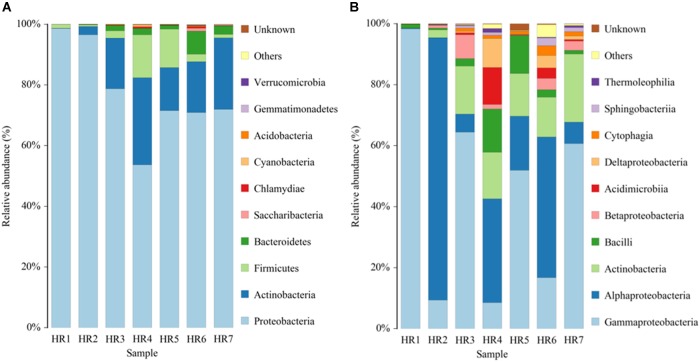
Relative abundances of bacterial communities at the phylum **(A)** and class **(B)** levels in different plant root samples, as determined by culture-independent analyses.

**FIGURE 5:**
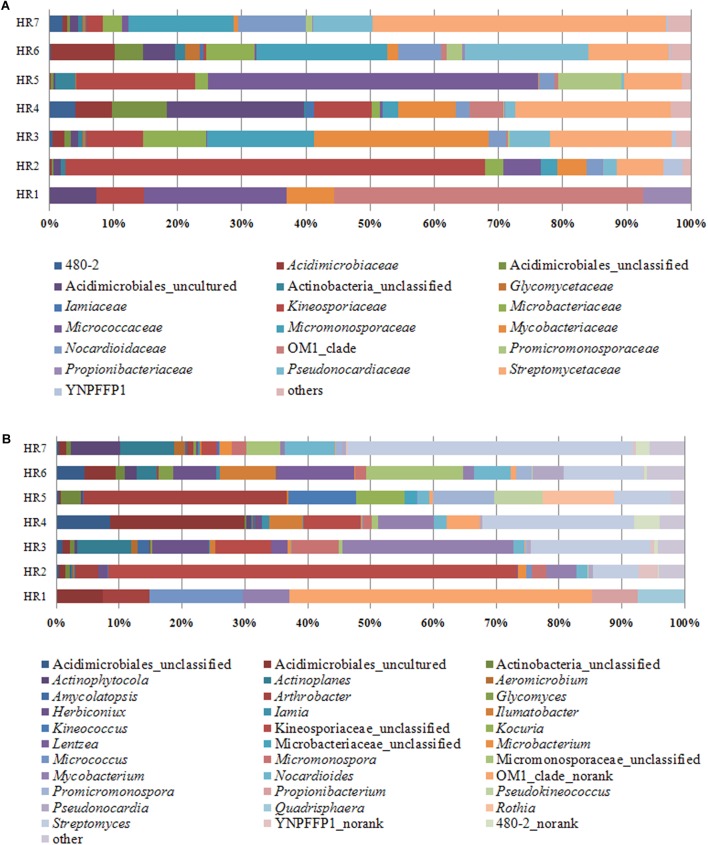
Relative abundances of bacteria at the actinobacterial family **(A)** and genus **(B)** levels in different plant root samples, as determined by culture-independent analyses.

**FIGURE 6:**
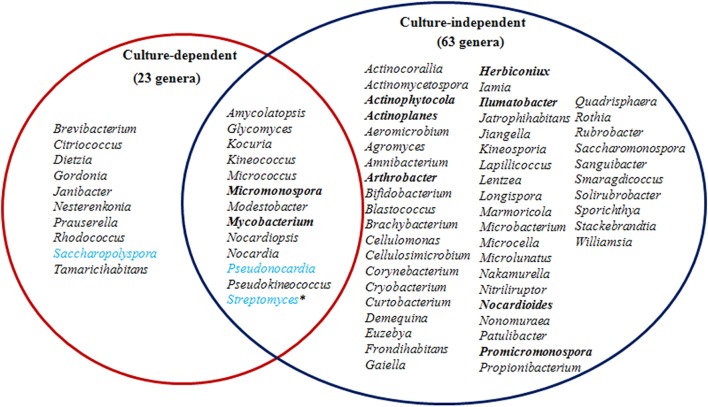
A comparison of endophytic actinobacterial genera detected in roots using culture-dependent and -independent methods. The most abundant genera detected by culture-independent analysis are indicated in bold and for the culture-dependent analysis are indicated in light blue. Asterisks indicate that the genus was abundant using both methodologies.

Taxonomic assignment of OTUs revealed the presence of 12 phyla, wherein the Proteobacteria were the most abundant in all samples, followed by the Actinobacteria, Firmicutes, and Bacteroidetes. Actinobacterial relative abundances ranged between 0.04 and 28.66% in each sample. The phylum was in lowest abundance in *Phragmites australis* (HR1) roots (0.04%), and highest in *Metaplexis japonica* (HR4) and *Spartina alterniflora* (HR7) roots, comprising 28.66 and 23.63% of the communities, respectively (Figure 4A). At the class level, most of the endophytic bacteria belonged to the *Gammaproteobacteria*, *Alphaproteobacteria*, *Actinobacteria*, *Bacilli*, *Betaproteobacteria*, and *Acidimicrobiia* clades (Figure 4B). Four classes of Actinobacteria were detected, including the *Actinobacteria*, *Acidimicrobiia*, *Nitriliruptoria*, and *Thermoleophilia*, of which *Actinobacteria* and *Acidimicrobiia* were the most abundant. In addition, 14 known actinobacterial orders were detected, with *Acidimicrobiales*, *Streptomycetales*, and *Micrococcales* comprising the most abundant orders. A total of 35 known actinobacterial families were also detected, with variation of families among samples (Figure 5A). At the genus level, 63 actinobacterial genera were observed, with *Streptomyces*, *Nocardioides*, *Mycobacterium*, *Micromonospora*, *Ilumatobacter*, *Herbiconiux*, *Arthrobacter*, and *Actinoplanes* representing the most abundant taxa within root tissues, but with varying abundances among samples. For example, *Streptomyces* was most abundant in the HR3, HR4, HR6, and HR7 samples while *Arthrobacter* was most abundant in the HR5 sample. The genera *Arthrobacter*, *Mycobacterium*, and *Micrococcus* were observed in all samples. Most root tissues harbored numerous actinobacterial genera (>30), and the HR7 sample contained the highest diversity (47 genera), while the HR1 and HR2 samples only harbored 4 and 21 genera, respectively. In addition to characterized taxa, a high proportion of populations classified as *Acidimicrobiales*, *Kineosporiaceae*, and *Micromonosporaceae* could not be assigned to a known genus (Figure 5B), indicating the presence of numerous novel taxa. HTS cultivation-independent analyses clearly led to the detection of more diverse endophytic Actinobacteria than did the cultivation-based methods. Of the 63 genera detected by cultivation-independent sequencing, 11 genera were also detected by cultivation, and *Streptomyces* were abundant among the communities identified using both methods (Figure 6).

### Antifungal and Fibrinolytic Activities of Isolates

Twenty-four of the 79 strains (30.4%) exhibiting antifungal activity against at least one of the pathogenic fungi that were tested. Twelve strains (15.2%) inhibited half of the pathogens that were tested, while eight strains exhibited even wider inhibition spectra. Two *Streptomyces* strains (KLBMP 1445 and KLBMP 1357), one *Pseudonocardia* strain (KLBMP 1272), and one *Saccharopolyspora* strain (KLBMP 1287) showed antifungal activity against a total of six to eight pathogens. The antifungal activity against *Marssonina brunnea* YH1 was by far the most prevalent (18 isolates, 22.8%) (Supplementary Table S1). Overall, six different genera exhibited antifungal activities, including the *Streptomyces* (10 isolates), *Pseudonocardia* (seven isolates), *Saccharopolyspora* (four isolates), *Prauserella* (one isolate), *Janibacter* (one isolate), and *Rhodococcus* (one isolate). We found that 40.5% (32 strains) of the representative strains showed fibrinolytic activities, which were distributed into nine genera, including *Glycomyces*, *Saccharopolyspora*, *Streptomyces*, *Amycolatopsis*, *Pseudonocardia*, *Tamaricihabitans*, *Nocardiopsis*, *Micromonospora*, and *Kocuria* (Table 2). However, 31.9% of the active strains only displayed weak fibrinolytic activity. Two *Glycomyces* strains (KLBMP 1269 and KLBMP 1483), two *Saccharopolyspora* strains (KLBMP 1270 and KLBMP 1337), and two *Streptomyces* strains (KLBMP 1361 and KLBMP 1469) showed highest fibrinolytic activity (Supplementary Table S2).

### Detection of Natural Product Biosynthesis Gene

A total of 34 of the 79 strains (43%) exhibited the presence of one or more types of natural product biosynthetic genes (PKS I, PKS II, NRPS, and Halo). NRPS genes were most frequently detected (16 strains; 20.3%), followed by Halo (12 strains; 15.2%). PKS I and PKS II genes were only detected in five and four of the 79 strains, respectively. The strains exhibiting the presence of biosynthetic genes were distributed among diverse genera (Table 2). The presence of PKS I genes was only observed in strains from the *Saccharopolyspora* and *Streptomyces* genera. In contrast, strains harboring NRPS and Halo genes were extensively distributed among nine and eight different genera, respectively. Both NRPS and Halo genes were only observed in five strains within the genus *Streptomyces*. Two strains, *Saccharopolyspora* sp. KLBMP 1333 and *Streptomyces* sp. KLBMP 1357, both exhibited the presence of two genes (PKS I and NRPS for KLBMP 1333, PKS II, and NRPS for KLBMP 1357; Supplementary Table S1). The positive amplification products of biosynthetic genes of some representative strains were sequenced, and the results confirmed that the amplification of these genes indeed encode the corresponding biosynthetic enzymes (data not shown).

## Discussion

Diverse endophytic actinomycetes have been found in many crops, model plants (e.g., *Arabidopsis thaliana*), and other plants from numerous typical environments (Conn and Franco, 2003; Qin et al., 2009; Golinska et al., 2015; Dinesh et al., 2017). The method used to isolate endophytic actinobacteria is an important factor that affects the acquisition and diversity of pure cultures. For example, plant surface sterilization, use of different selective media types, and plant treatment methods can all influence cultivation-dependent outcomes. In this study, we used a previously described five-step sterile sample processing protocol, longer incubation times, and various selective media to obtain pure endophytic actinobacterial cultures (Qin et al., 2009). Starch-casein agar and sodium propionate agar that were supplemented with 3% (w/v) NaCl yielded the greatest diversity of endophytic actinobacterial isolates, which comprised 10 different genera, including those that have rarely been reported as endophytes such as *Glycomyces*, *Modestobacter*, and *Pseudokineococcus* (Table 1). Starch-casein agar has previously been suggested to be effective in the isolation of Actinobacteria from saline environments (Abbas, 2006). Similarly, sodium propionate medium is seen to be effective for isolating endophytic actinobacteria from various plant hosts (Qin et al., 2009; Li et al., 2012). Our recent investigation also found that these two types of media supplemented with 3% NaCl were suitable for isolating rare actinomycetes from rhizosphere soils associated with coastal halophytes (Gong et al., 2018). CMC medium has also been suggested as suitable for isolating rare endophytic actinobacteria (Kaewkla and Franco, 2013). Although only eight strains were obtained in this study using the CMC medium, they were distributed among five genera, which included *Dietzia*, *Glycomyces*, and *Kocuria*, and are all rarely reported as endophytes. These results suggest that further investigations of endophytic actinobacteria should strongly consider the in-depth exploration of the cultivation methods that are to be employed.

Marine environments host a diversity of rare actinobacteria (Pennings and Bertness, 2001; Hassan et al., 2017). Because coastal salt marshes are characterized by both terrestrial and marine characteristics, abundant and diverse endophytic actinobacterial resources are likely present in these environments. A total of 23 different actinobacterial genera were identified in the present study, including many novel species, suggesting a high diversity of actinobacteria within the native plants of the salt marsh investigated here. To our knowledge, such high diversity of culturable endophytic actinobacteria is yet to be demonstrated from such habitats. Tian and Zhang (2017) analyzed the endophytic and rhizosphere bacterial diversity of the Chinese coastal halophyte *Messerschmidia sibirica* using high-throughput sequencing method, and they revealed that the relative abundance of the phylum *Actinobacteria* accounts for more than 6.2% across all leaf, stem, and root tissues. Yamamoto et al. (2018) also found that *Actinobacteria* in the coastal halophyte *Glaux maritima* root tissues collected from the eastern part of Japan was extremely high using the Illumina MiSeq sequencing technique. However, none of these studies reported pure culture isolation of endophytic actinobacteria and their diversity. Recently, Jiang et al. (2018) obtained diverse endophytic actinobacterial strains (101 isolates from 15 families and 28 genera) from five mangrove plants collected from the Guangxi Zhuang Autonomous Region of China. Taken together with the above results, our study confirms that coastal plants contain abundant and diverse endophytic actinomycete resources. Interestingly, in this study, most of the culturable endophytic actinobacterial genera, such as *Streptomyces*, *Saccharopolyspora*, *Pseudonocardia* and *Micromonospora*, were also successfully cultured from their corresponding host plants rhizosphere soils previously (Gong et al., 2018). This result supports the previous conclusion that rhizosphere microorganisms are the main origin of endophytic bacteria (Mora-Ruiz Mdel et al., 2016).

A total of 72 known actinobacterial genera were detected from seven root samples using a combination of cultivation-dependent and -independent techniques (Figure 6). To our knowledge, this is the first investigation of coastal plant-associated endophytic actinobacterial diversity using both cultivation- and culture-independent methods. The majority of the isolates were obtained from plant roots (Table 2), which is consistent with the results of other studies, indicating that endophytic actinomycetes primarily colonized root tissues (Qin et al., 2009; Kaewkla and Franco, 2013; Tian and Zhang, 2017). The community composition of actinobacterial taxa at the phylum, family, and genus levels varied among different root samples (Figure 4, 5). Recent investigations of plant microbiomes have suggested that plant genotypes significantly affect the composition of symbiotic root microbiota (Wintermans et al., 2016; Li et al., 2018). Therefore, the root-associated actinobacterial compositional differences observed here could be explained by differences in host plant species. At the genus level of actinomycetes, differences were observed in the number of genera detected by cultivation-dependent and -independent methods (Figure 6). Specifically, high-throughput 16S rRNA gene sequencing resulted in the detection of 63 known genera, including many genera that have rarely been reported as endophytes such as *Herbiconiux*, *Actinoplanes*, *Ilumatobacter*, and *Actinophytocola* (Pérez et al., 2016). Furthermore, HTS within root samples resulted in the detection of much higher levels of taxa, which could not be classified into known taxonomic groups. These results indicated that coastal salt marsh plants harbored a high level of rare endophytic actinobacteria and novel taxa. Only 11 of the 63 genera detected by 16S rRNA gene sequencing were also detected by cultivation, while nine cultivated genera were not detected by sequence analysis (Figure 6). However, some genera that are widely distributed among different environments and that display diverse ecological functions associated with plants, including *Arthrobacter* (Prum et al., 2018; Qin et al., 2018), were not detected by cultivation. This result highlights the impetus to continue developing cultivation methods and strategies to culture greater diversity in future investigations. These disparities in the detected taxa could potentially be explained by the variable efficiency of genomic DNA extraction among species, taxonomic biases of primers used for PCR amplification, and the recalcitrance of certain taxa to cultivation under laboratory conditions. These explanations are consistent with the previous reports (Qin et al., 2012; Chen et al., 2016; Eevers et al., 2016). Therefore, it is critical to use a combined cultivation-dependent and -independent approach to accurately evaluate the composition of endophytic actinobacterial communities.

*Streptomyces* are the most prevalent genus observed among endophytic actinobacteria (Conn and Franco, 2003; Kaewkla and Franco, 2013; Qin et al., 2015b; Dinesh et al., 2017; Passari et al., 2017). In addition, many rare endophytic actinomycetes have also been recently reported (Qin et al., 2011; Dinesh et al., 2017). Our observation that *Streptomyces* were the most dominant species within coastal salt marsh plants is consistent with these previous studies. Endophytic *Streptomyces* spp. are well-known for their capacity to produce many types of bioactive metabolites, and several species have been reported as beneficial toward plant development and health (Mitra et al., 2008; Vurukonda et al., 2018). The highest proportions of secondary metabolite biosynthetic genes observed in this study were in *Streptomyces* spp. strains (63.2%), with at least 12 strains harboring PKS, NRPS, and/or Halo genes (Table 2). This indicates that the strains screened possess diverse secondary metabolite gene clusters, as evidenced by the presence of these genes. PCR-based screening of biosynthetic genes is a useful and efficient method to conduct bioprospecting of certain type of natural products producing actinobacteria. For example, based on the FADH_2_-dependent halogenase genes screening from the mangrove-derived actinomycetes, Li et al. (2013) obtained a new enduracidin producer, *Streptomyces atrovirens* MGR140, which was further identified and confirmed by gene disruption and HPLC analysis. The presence of halo genes here is particular interesting, because more than 4,700 halogenated compounds have been reported, which are important sources for new drugs, due to their high diversity in structure and activity (Liao et al., 2016). Moreover, *Streptomyces* strains exhibited significant antagonistic activities toward fungal pathogens, with 52.6% strains displaying antifungal activities against at least one of the pathogentic fungi that were tested. One isolate, KLBMP 1357, exhibited broad-spectrum antifungal activity and contained both the PKS II and NRPS genes (Supplementary Table S1), meriting further research of its bioactive secondary metabolites. Nevertheless, antifungal activities and the presence of certain secondary metabolite functional genes did not appear to be directly correlated in this study. For example, functional secondary metabolite genes were not detected in the *Pseudonocardia* strain KLBMP 1272, despite the fact that it exhibited broad-spectrum antifungal activity. This result could potentially be due to one of two reasons. First, the fermentation medium used to cultivate the strain may not promote the production of antifungal secondary metabolites. Second, the degenerate primer pairs that were used to amplify the functional genes may not be suitable for amplification of these genes within certain strains. Alternatively, the strains considered here may actually lack the biosynthetic genes tested, but possess other secondary metabolite biosynthetic genes (Schneemann et al., 2010). Moreover, we have only carried out antifungal screening, and the results of gene screening may be more relevant to antibacterial and anti-tumor activity. The results reported here are consistent with those from a recent screening of culturable actinomycetes from hot springs, lichens, and tea plants (Liu et al., 2016, 2017, Wei et al., 2018). Considering that many actinomycetes were isolated from root tissues, and showed strong antagonistic activity against plant pathogenic fungi, we may speculate that these endophytic actinomycetes have potential for biological control and potential contribution to their ecological adaptation within roots.

We also observed that some strains of *Pseudonocardia* and *Saccharopolyspora* that are rarely described as endophytes exhibited good antifungal activity, including strains KLBMP 1272, KLBMP 1413, and KLBMP 1287. *Pseudonocardia* have been previously observed as dominant endophytes within plants; in addition, some new endophytic species have been discovered recently (Qin et al., 2009; Li et al., 2012; Zhao et al., 2012). In contrast, endophytic *Saccharopolyspora* spp. isolates have been rarely reported (Qin et al., 2008; Li et al., 2009). Recently, endophytic *Pseudonocardia* isolates were identified that were associated with mangrove trees and sea cucumbers. The isolates displayed antimicrobial and cytotoxic activities, and subsequently yielded novel bioactive compounds (Mangamuri et al., 2015; Ye et al., 2016). Microorganism is an important source of thrombolytic agents for thrombosis treatment, and lots of fibrinolytic enzymes were successively discovered from different microorganisms, including marine actinomycetes (Verma et al., 2018). However, non-*Streptomyces* rare actinobacteria and endophytes origin fibrinolytic enzymes were rarely reported (Lu et al., 2010; Mohanasrinivasan et al., 2017). Thus, we carried out the fibrinolytic activity screening for the endophytic actinobacterial isolates. Interestingly, we found that more than 40% of the strains secreted extracellular proteases that exhibited fibrinolytic activity in this study. It is noteworthy that some new species (KLBMP 1269, KLBMP 1483, KLBMP 1305, and KLBMP 1278) displayed strong and moderate fibrinolytic activity (Supplementary Table S2), meriting further purification and characterization of their fibrinolytic enzymes and exploring the thrombolytic effect. In addition, to our knowledge, this is the first time that strains of endophytic actinobacteria and the rare actinobacteria genera *Glycomyces*, *Saccharopolyspora*, *Amycolatopsis*, *Pseudonocardia*, and *Tamaricihabitans* have been reported to have fibrinolytic activity. Taken together, these results indicate the coastal marsh halophytes-associated endophytic actinobacterial yet-to-be discovered bioactive metabolites and functions warrant further exploration.

## Conclusion

Endophytic actinobacterial community structure was analyzed among coastal salt marsh halophyte plants within the Jiangsu Province of Eastern China using both culture-dependent and -independent methods. Coastal salt marsh plants harbored abundant and diverse communities of endophytic actinobacteria, including many rare actinobacteria and novel taxa. These results also indicate that halophyte plants have an important role in shaping the community composition of endophytic microbiota within roots. Taken together, the results of this study suggest that coastal salt marsh plants-associated endophytic actinobacteria represent a promising, yet underexplored, new resource for discovering bioactive natural products with potential as biocontrol agents and for fibrinolytic enzyme production.

## Author Contributions

CZ, KX, and SQ designed the experiments. PC, XJ, YX, and SQ performed the experiments. PC, CZ, YX, and SQ analyzed the data and prepared the manuscript.

## Conflict of Interest Statement

The authors declare that the research was conducted in the absence of any commercial or financial relationships that could be construed as a potential conflict of interest.

## References

[B1] Abbas I. H. (2006). A biological and biochemical studies of actinomycetes isolated from Kuwait saline soil-Kuwait. *J. Appl. Sci. Res.* 2 809–815.

[B2] Álvarez-Pérez J. M. González-García S. Cobos R. Olego M. Á Ibañez A. Díez-Galán A. (2017). Use of endophytic and rhizosphere actinobacteria from grapevine plants to reduce nursery fungal graft infections that lead to young grapevine decline. *Appl. Environ. Microbiol.* 83:e1564–17. 10.1128/AEM.01564-17 28986378 PMC5717199

[B3] Astrup T. Müllertz S. (1952). The fibrin plate method for estimating fibrinolytic activity. *Arch. Biochem. Biophys.* 40 346–351. 12997222 10.1016/0003-9861(52)90121-5

[B4] Ayuso-Sacido A. Genilloud O. (2005). New PCR primers for the screening of NRPS and PKS-I systems in actinomycetes: detection and distribution of these biosynthetic gene sequences in major taxonomic groups. *Microb. Ecol.* 49 10–24. 15614464 10.1007/s00248-004-0249-6

[B5] Bai J. L. Wang Y. Qin S. Ding P. Xing K. Yuan B. (2016). *Nocardia jiangsuensis* sp. nov., an actinomycete isolated from coastal soil. Int. J. Syst. Evol. Microbiol. 66 4633–4638. 10.1099/ijsem.0.001402 27503503

[B6] Bèrdy J. (2005). Bioactive microbial metabolites. *J. Antibiot.* 58 1–26. 15813176 10.1038/ja.2005.1

[B7] Bian G. K. Feng Z. Z. Qin S. Xing K. Wang Z. Cao C. L. (2012). *Kineococcus endophytica* sp. nov., a novel endophytic actinomycete isolated from a coastal halophyte in Jiangsu, China. *Antonie van Leeuwenhoek.* 102 621–628. 10.1007/s10482-012-9757-4 22669199

[B8] Caporaso J. G. Kuczynski J. Stombaugh J. Bittinger K. Bushman F. D. Costello E. K. (2010). QIIME allows analysis of high-throughput community sequencing data. *Nat. Methods* 7 335–336. 20383131 10.1038/nmeth.f.303 PMC3156573

[B9] Chelius M. K. Triplett E. W. (2001). The diversity of archaea and bacteria in association with the roots of *Zea mays* L. *Microb. Ecol.* 41 252–263. 11391463 10.1007/s002480000087

[B10] Chen P. Zhang L. Guo X. Dai X. Liu L. Xi L. (2016). Diversity, biogeography, and biodegradation potential of actinobacteria in the deep-sea sediments along the southwest Indian ridge. *Front. Microbiol.* 7:1340. 10.3389/fmicb.2016.01340 27621725 PMC5002886

[B11] Chun J. Oren A. Ventosa A. Christensen H. Arahal D. R. da Costa M. S. (2018). Proposed minimal standards for the use of genome data for the taxonomy of prokaryotes. *Int. J. Syst. Evol. Microbiol.* 68 461–466. 10.1099/ijsem.0.002516 29292687

[B12] Conn V. M. Franco C. M. (2003). Isolation and identification of actinobacteria from surface-sterilized wheat roots. *Appl. Environ. Microbiol.* 69 5603–5608. 12957950 10.1128/AEM.69.9.5603-5608.2003 PMC194995

[B13] Conn V. M. Walker A. R. Franco C. M. (2008). Endophytic actinobacteria induce defense pathways in *Arabidopsis thaliana*. *Mol. Plant Microbe. Interact.* 21 208–218. 10.1094/MPMI-21-2-0208 18184065

[B14] Dinesh R. Srinivasan V. Sheeja T. E. Anandaraj M. Srambikkal H. (2017). Endophytic actinobacteria: diversity, secondary metabolism and mechanisms to unsilence biosynthetic gene clusters. *Crit. Rev. Microbiol.* 43 546–566. 10.1080/1040841X.2016.1270895 28358596

[B15] Ding P. Bai J. L. Wang T. T. Sun Y. Cao C. L. Jiang J. H. (2018). *Nocardia rhizosphaerihabitans* sp. nov., a novel actinomycete isolated from a coastal soil. Int. J. Syst. Evol. Microbiol. 68 192–197. 10.1099/ijsem.0.002481 29125460

[B16] Du X. Zhai Y. Deng Q. Tan H. Cao L. (2018). Illumina-based sequencing analysis directed selection for actinobacterial probiotic candidates for banana plants. *Probiotics. Antimicrob. Proteins* 10 284–292. 10.1007/s12602-017-9293-7 28560514

[B17] Edgar R. C. (2013). UPARSE: highly accurate OTU sequences from microbial amplicon reads. *Nat. Methods* 10 996–998. 10.1038/nmeth.2604 23955772

[B18] Edgar R. C. Haas B. J. Clemente J. C. Quince C. Knight R. (2011). UCHIME improves sensitivity andspeed of chimera detection. *Bioinformatics* 27 2194–2200. 10.1093/bioinformatics/btr381 21700674 PMC3150044

[B19] Eevers N. Beckers B. Op de Beeck M. White J. C. Vangronsveld J. Weyens N. (2016). Comparison between cultivated and total bacterial communities associated with *Cucurbita pepo* using cultivation-dependent techniques and 454 pyrosequencing. *Syst. Appl. Microbiol.* 39 58–66. 10.1016/j.syapm.2015.11.001 26656884

[B20] Gao P. Huang Y. (2009). Detection, distribution, and organohalogen compound discovery implications of the reduced flavin adenine dinucleotide-dependent halogenase gene in major filamentous actinomycete taxonomic groups. *Appl. Environ. Microbiol.* 75 4813–4820. 10.1128/AEM.02958-08 19447951 PMC2708417

[B21] Golinska P. Wypij M. Agarkar G. Rathod D. Dahm H. Rai M. (2015). Endophytic actinobacteria of medicinal plants: diversity and bioactivity. *Antonie van Leeuwenhoek* 108 267–289. 26093915 10.1007/s10482-015-0502-7 PMC4491368

[B22] Gong Y. Bai J. L. Yang H. T. Zhang W. D. Xiong Y. W. Ding P. (2018). Phylogenetic diversity and investigation of plant growth-promoting traits of actinobacteria in coastal salt marsh plant rhizospheres from Jiangsu. China. *Syst. Appl. Microbiol.* 41 516–527. 10.1016/j.syapm.2018.06.003 29934111

[B23] Hamedi J. Mohammadipanah F. Ventosa A. (2013). Systematic and biotechnological aspects of halophilic and halotolerant actinomycetes. *Extremophiles* 17 1–13. 10.1007/s00792-012-0493-5 23129307

[B24] Hassan S. S. Anjum K. Abbas S. Q. Akhter N. Shagufta B. I. Shah S. A. (2017). Emerging biopharmaceuticals from marine actinobacteria. *Environ. Toxicol. Pharmacol.* 49 34–47. 10.1016/j.etap.2016.11.015 27898308

[B25] Hong K. Gao A. H. Xie Q. Y. Gao H. Zhuang L. Lin H. P. (2009). Actinomycetes for marine drug discovery isolated from mangrove soils and plants in China. *Mar. Drugs* 7 24–44. 10.3390/md7010024 19370169 PMC2666887

[B26] Hornung A. Bertazzo M. Dziarnowski A. Schneider K. Welzel K. Wohlert S. E. (2007). A genomic screening approach to the structure-guided identification of drug candidates from natural sources. *Chembiochem* 8 757–766. 17407125 10.1002/cbic.200600375

[B27] Jiang Z. K. Tuo L. Huang D. L. Osterman I. A. Tyurin A. P. Liu S. W. (2018). Diversity, novelty, and antimicrobial activity of endophytic actinobacteria from mangrove plants in Beilun Estuary national nature reserve of Guangxi. China. *Front. Microbiol.* 9:868. 10.3389/fmicb.2018.00868 29780376 PMC5945994

[B28] Kaewkla O. Franco C. M. (2013). Rational approaches to improving the isolation of endophytic actinobacteria from Australian native trees. *Microb. Ecol.* 65 384–393. 10.1007/s00248-012-0113-z 22976339

[B29] Kim O. S. Cho Y. J. Lee K. Yoon S. H. Kim M. Na H. (2012). Introducing EzTaxon-e: a prokaryotic 16S rRNA gene sequence database with phylotypes that represent uncultured species. *Int. J. Syst. Evol. Microbiol.* 62 716–721. 10.1099/ijs.0.038075-0 22140171

[B30] Lechevalier M. P. Lechevalier H. A. (1980). “The chemotaxonomy of actinomycetes,” in *Actinomycete Taxonomy*, eds Dietz A. Thayer D. W. (Arlington, VA: Society for Industrial Microbiology), 22–291.

[B31] Li J. Zhao G. Z. Huang H. Y. Qin S. Zhu W. Y. Zhao L. X. (2012). Isolation and characterization of culturable endophytic actinobacteria associated with *Artemisia annua* L. *Antonie van Leeuwenhoek* 101 515–527. 10.1007/s10482-011-9661-3 22038129

[B32] Li J. Zhao G. Z. Qin S. Huang H. Y. Zhu W. Y. Xu L. H. (2009). *Saccharopolyspora tripterygii* sp. nov., an endophytic actinomycete isolated from the stem of *Tripterygium hypoglaucum*. Int. J. Syst. Evol. Microbiol. 59 3040–3044. 10.1099/ijs.0.011734-0 19643898

[B33] Li W. J. Xu P. Schumann P. Zhang Y. Q. Pukall R. Xu L. H. (2007). *Georgenia ruanii* sp. nov., a novel actinobacterium isolated from forest soil in Yunnan (China) and emended description of the genus Georgenia. *Int. J. Syst. Evol. Microbiol.* 57 1424–1428. 17625169 10.1099/ijs.0.64749-0

[B34] Li X. G. Tang X. M. Xiao J. Ma G. H. Xu L. Xie S. J. (2013). Harnessing the potential of halogenated natural product biosynthesis by mangrove-derived actinomycetes. *Mar. Drugs* 11 3875–3890. 10.3390/md11103875 24129229 PMC3826140

[B35] Li Y. Wu X. Chen T. Wang W. Liu G. Zhang W. (2018). Plant phenotypic traits eventually shape its microbiota: a common garden test. *Front. Microbiol.* 9:2479. 10.3389/fmicb.2018.02479 30459725 PMC6232875

[B36] Liao L. Chen R. Jiang M. Tian X. Liu H. Yu Y. (2016). Bioprospecting potential of halogenases from Arctic marine actinomycetes. *BMC Microbiol.* 16:34. 10.1186/s12866-016-0662-2 26964536 PMC4785625

[B37] Liu C. Jiang Y. Wang X. Chen D. Chen X. Wang L. (2017). Diversity, antimicrobial activity, and biosynthetic potential of cultivable actinomycetes associated with lichen symbiosis. *Microb. Ecol.* 74 570–584. 10.1007/s00248-017-0972-4 28361265

[B38] Liu L. Salam N. Jiao J. Y. Jiang H. C. Zhou E. M. Yin Y. R. (2016). Diversity of culturable thermophilic actinobacteria in hot springs in Tengchong, China and studies of their biosynthetic gene profiles. *Microb. Ecol.* 72 150–162. 10.1007/s00248-016-0756-2 27048448

[B39] Long H. H. Sonntag D. G. Schmidt D. D. Baldwin I. T. (2010). The structure of the culturable root bacterial endophyte community of *Nicotiana attenuata* is organized by soil composition and host plant ethylene production and perception. *New Phytol.* 185 554–567. 10.1111/j.1469-8137.2009.03079.x 19906091

[B40] Lu F. Lu Z. Bie X. Yao Z. Wang Y. Lu Y. (2010). Purification and characterization of a novel anticoagulant and fibrinolytic enzyme produced by endophytic bacterium *Paenibacillus polymyxa* EJS-3. *Thromb Res.* 126 e349–e355. 10.1016/j.thromres.2010.08.003 20813399

[B41] Mangamuri U. K. Vijayalakshmi M. Poda S. Manavathi B. Bhujangarao C. H. Venkateswarlu Y. (2015). Bioactive metabolites produced by *Pseudonocardia* endophytica VUK-10 from mangrove sediments: isolation, chemical structure determination and bioactivity. *J. Microbiol. Biotechnol.* 25 629–636. 25418482 10.4014/jmb.1407.07041

[B42] Metsä-Ketelä M. Salo V. Halo L. Hautala A. Hakala J. Mäntsälä P. (1999). An efficient approach for screening minimal PKS genes from *Streptomyces*. *FEMS Microbiol. Lett.* 180 1–6. 10547437 10.1111/j.1574-6968.1999.tb08770.x

[B43] Miao G. P. Zhu C. S. Feng J. T. Han L. R. Zhang X. (2015). Effects of plant stress signal molecules on the production of wilforgine in an endophytic actinomycete isolated from *Tripterygium wilfordii* Hook.f. *Curr. Microbiol.* 70 571–579. 10.1007/s00284-014-0758-6 25523369

[B44] Mitra A. Santra S. C. Mukherjee J. (2008). Distribution of actinomycetes, their antagonistic behaviour and the physico-chemical characteristics of the world’s largest tidal mangrove forest. *Appl. Microbiol. Biotechnol.* 80 685–695. 18679673 10.1007/s00253-008-1626-8

[B45] Mohanasrinivasan V. Yogesh S. Govindaraj A. Naine S. J. Devi C. S. (2017). In vitro thrombolytic potential of actinoprotease from marine *Streptomyces violaceus* VITYGM. *Cardiovasc*. *Hematol. Agents Med. Chem.* 14 120–124. 10.2174/1871525715666161104112553 27823551

[B46] Mora-Ruiz Mdel R. Font-Verdera F. Orfila A. Rita J. Rosselló-Móra R. (2016). Endophytic microbial diversity of the halophyte *Arthrocnemum macrostachyum* across plant compartments. *FEMS Microbiol. Ecol.* 92:fiw145. 10.1093/femsec/fiw145 27353659

[B47] Passari A. K. Mishra V. K. Singh G. Singh P. Kumar B. Gupta V. K. (2017). Insights into the functionality of endophytic actinobacteria with a focus on their biosynthetic potential and secondary metabolites production. *Sci. Rep.* 7:11809. 10.1038/s41598-017-12235-4 28924162 PMC5603540

[B48] Pennings S. C. Bertness M. D. (2001). “Salt marsh communities,” in *Marine Community Ecology*, eds Bertness M. D. Gaines S. D. Hay M. E. (Sunderland, MA: Sinauer Associates, Inc.).

[B49] Pérez M. L. Collavino M. M. Sansberro P. A. Mroginski L. A. Galdeano E. (2016). Diversity of endophytic fungal and bacterial communities in *Ilex paraguariensis* grown under field conditions. *World J. Microbiol. Biotechnol.* 32:61. 10.1007/s11274-016-2016-5 26925623

[B50] Prum C. Dolphen R. Thiravetyan P. (2018). Enhancing arsenic removal from arsenic-contaminated water by *Echinodorus cordifolius*-endophytic *Arthrobacter creatinolyticus* interactions. *J. Environ. Manage.* 213 11–19. 10.1016/j.jenvman.2018.02.060 29477846

[B51] Purushotham N. Jones E. Monk J. Ridgway H. (2018). Community structure of endophytic actinobacteria in a New Zealand native medicinal plant *Pseudowintera colorata* (Horopito) and their influence on plant growth. *Microb. Ecol.* 76 729–740. 10.1007/s00248-018-1153-9 29435598

[B52] Qin S. Bai J. L. Wang Y. Feng W. W. Yuan B. Sun Y. (2015a). *Tamaricihabitans halophyticus* gen. nov., sp. nov., an endophytic actinomycete of the family *Pseudonocardiaceae*. *Int. J. Syst. Evol. Microbiol.* 65 4662–4668. 10.1099/ijsem.0.000628 26410726

[B53] Qin S. Miao Q. Feng W. W. Wang Y. Zhu X. Xing K. (2015b). Biodiversity and plant growth promoting traits of culturable endophytic actinobacteria associated with *Jatropha curcas* L. growing in Panxi dry-hot valley soil. *Appl. Soil. Ecol.* 93 47–55.

[B54] Qin S. Bian G. K. Tamura T. Zhang Y. J. Zhang W. D. Cao C. L. (2013a). *Streptomyces halophytocola* sp. nov., an endophytic actinomycete isolated from the surface-sterilized stems of a coastal halophyte *Tamarix chinensis* Lour. *Int. J. Syst. Evol. Microbiol.* 63 2770–2775. 10.1099/ijs.0.047456-0 23291896

[B55] Qin S. Bian G. K. Zhang Y. J. Xing K. Cao C. L. Liu C. H. (2013b). *Modestobacter roseus* sp. nov., an endophytic actinomycete isolated from the coastal halophyte *Salicornia europaea* Linn., and emended description of the genus *Modestobacter*. *Int. J. Syst. Evol. Microbiol.* 63 2197–2202. 10.1099/ijs.0.044412-0 23148095

[B56] Qin S. Chen H. H. Zhao G. Z. Li J. Zhu W. Y. Xu L. H. (2012). Abundant and diverse endophytic actinobacteria associated with medicinal plant *Maytenus austroyunnanensis* in Xishuangbanna tropical rainforest revealed by culture-dependent and culture-independent methods. *Environ. Microbiol. Rep.* 4 522–531. 10.1111/j.1758-2229.2012.00357.x 23760897

[B57] Qin S. Feng W. W. Wang T. T. Ding P. Xing K. Jiang J. H. (2017). Plant growth-promoting effect and genomic analysis of the beneficial endophyte *Streptomyces* sp. KLBMP 5084 isolated from halophyte *Limonium sinense*. *Plant Soil* 416 117–132.

[B58] Qin S. Feng W. W. Zhang Y. J. Wang T. T. Xiong Y. W. Xing K. (2018). Diversity of bacterial microbiota of coastal halophyte *Limonium sinense* and amelioration of salinity stress damage by symbiotic plant growth-promoting actinobacterium *Glutamicibacter halophytocola* KLBMP 5180. *Appl. Environ. Microbiol.* 84:e1533–18. 10.1128/AEM.01533-18 30054358 PMC6146988

[B59] Qin S. Li J. Chen H. H. Zhao G. Z. Zhu W. Y. Jiang C. L. (2009). Isolation, diversity, and antimicrobial activity of rare actinobacteria from medicinal plants of tropical rain forests in Xishuangbanna. China. *Appl. Environ. Microbiol.* 75 6176–6186. 10.1128/AEM.01034-09 19648362 PMC2753051

[B60] Qin S. Li J. Zhao G. Z. Chen H. H. Xu L. H. Li W. J. (2008). *Saccharopolyspora endophytica* sp. nov., an endophytic actinomycete isolated from the root of *Maytenus austroyunnanensis*. *Syst. Appl. Microbiol.* 31 352–357. 10.1016/j.syapm.2008.08.001 18929936

[B61] Qin S. Li W. J. Dastager S. G. Hozzein W. N. (2016). Editorial: actinobacteria in special and extreme habitats: diversity, function roles, and environmental adaptations. *Front. Microbiol.* 7:1415. 10.3389/fmicb.2016.01415 27660627 PMC5014857

[B62] Qin S. Xing K. Jiang J. H. Xu L. H. Li W. J. (2011). Biodiversity, bioactive natural products and biotechnological potential of plant-associated endophytic actinobacteria. *Appl. Microbiol. Biotechnol.* 89 457–473. 10.1007/s00253-010-2923-6 20941490

[B63] Qin S. Zhang Y. J. Yuan B. Xu P. Y. Xing K. Wang J. (2014). Isolation of ACC deaminase-producing habitat-adapted symbiotic bacteria associated with halophyte *Limonium sinense* (Girard) Kuntze and evaluating their plant growth-promoting activity under salt stress. *Plant Soil* 374 753–766.

[B64] Quast C. Pruesse E. Yilmaz P. Gerken J. Schweer T. Yarza P. (2013). The SILVA ribosomal RNA gene database project: improved data processing and web-based tools. *Nucleic Acids Res.* 41 D590–D596. 10.1093/nar/gks1219 23193283 PMC3531112

[B65] Reysenbach A. Pace N. (1995). “Reliable amplification of hyperthermophilic archaeal 16S rRNA genes by the polymerase chain reaction,” in *Archaea: A Laboratory Manual*, ed. Robb F. (Cold Spring Harbor, NY: Cold Spring Harbor Laboratory Press), 101–107.

[B66] Romero D. Pérez-García A. Rivera M. E. Cazorla F. M. de Vicente A. (2004). Isolation and evaluation of antagonist bacteria towards the cucurbit powdery mildew fungus *Podosphaera fusca*. *Appl. Microbiol. Biotechnol.* 64 263–269. 13680203 10.1007/s00253-003-1439-8

[B67] Schloss P. D. Westcott S. L. Ryabin T. Hall J. R. Hartmann M. Hollister E. B. (2009). Introducing mothur: open-source, platform-independent, community-supported software for describing and comparing microbial communities. *Appl. Environ. Microbiol.* 75 7537–7541. 10.1128/AEM.01541-09 19801464 PMC2786419

[B68] Schneemann I. Nagel K. Kajahn I. Labes A. Wiese J. Imhoff J. F. (2010). Comprehensive investigation of marine actinobacteria associated with the sponge *Halichondria panicea*. *Appl. Environ. Microbiol.* 76 3702–3714. 10.1128/AEM.00780-10 20382810 PMC2876447

[B69] Shirling E. B. Gottlieb D. (1966). Methods for characterization of *Streptomyces* species. *Int. J. Syst. Bacteriol.* 16 313–340.

[B70] Stackebrandt E. Frederiksen W. Garrity G. M. Grimont P. A. Kämpfer P. Maiden M. C. (2002). Report of the ad hoc committee for the reevaluation of the species definition in bacteriology. *Int. J. Syst. Evol. Microbiol.* 52 1043–1047. 12054223 10.1099/00207713-52-3-1043

[B71] Suga T. Kimura T. Inahashi Y. Iwatsuki M. Nonaka K. Také A. (2018). Hamuramicins A and B, 22-membered macrolides, produced by an endophytic actinomycete *Allostreptomyces* sp. K12-0794. *J. Antibiot.* 71 619–625. 10.1038/s41429-018-0055-x 29691484

[B72] Tamura K. Stecher G. Peterson D. Filipski A. Kumar S. (2013). MEGA6: molecular evolutionary genetics analysis version 6.0. *Mol. Biol. Evol.* 30 2725–2729. 10.1093/molbev/mst197 24132122 PMC3840312

[B73] Tian X. Cao L. Tan H. Han W. Chen M. Liu Y. (2007). Diversity of cultivated and uncultivated actinobacterial endophytes in the stems and roots of rice. *Microb. Ecol.* 53 700–707. 17334856 10.1007/s00248-006-9163-4

[B74] Tian X. Y. Zhang C. S. (2017). Illumina-based analysis of endophytic and rhizosphere bacterial diversity of the coastal halophyte *Messerschmidia sibirica*. *Front. Microbiol.* 8:2288. 10.3389/fmicb.2017.02288 29209296 PMC5701997

[B75] Trujillo M. E. Riesco R. Benito P. Carro L. (2015). Endophytic actinobacteria and the interaction of *Micromonospora* and nitrogen fixing Plants. *Front. Microbiol.* 6:1341 10.3389/fmicb.2015.01341 PMC4664631 26648923

[B76] Verma P. Chatterjee S. Keziah M. S. Devi S. C. (2018). Fibrinolytic protease from marine *Streptomyces rubiginosus* VITPSS1. *Cardiovasc. Hematol. Agents Med. Chem.* 16 44–55. 10.2174/1871525716666180226141551 29485011

[B77] Vurukonda S. S. K. P. Giovanardi D. Stefani E. (2018). Plant growth promoting and biocontrol activity of *Streptomyces* spp. as endophytes. *Int. J. Mol. Sci.* 19:E952. 10.3390/ijms19040952 29565834 PMC5979581

[B78] Wang Q. Garrity G. M. Tiedje J. M. Cole J. R. (2007). Naive Bayesian classifier for rapid assignment of rRNA sequences into the new bacterial taxonomy. *Appl. Environ. Microbiol.* 73 5261–5267. 17586664 10.1128/AEM.00062-07 PMC1950982

[B79] Wang Y. Liu W. Feng W. W. Zhou X. Q. Bai J. L. Yuan B. (2015). *Nocardia rhizosphaerae* sp. nov., a novel actinomycete isolated from the coastal rhizosphere of *Artemisia* Linn., China. *Antonie van Leeuwenhoek* 108 31–39. 10.1007/s10482-015-0460-0 25896308

[B80] Wei W. Zhou Y. Chen F. Yan X. Lai Y. Wei C. (2018). Isolation, diversity, and antimicrobial and immunomodulatory activities of endophytic actinobacteria from tea cultivars Zijuan and Yunkang-10 (*Camellia sinensis* var. *assamica)*. *Front. Microbiol.* 9:1304. 10.3389/fmicb.2018.01304 29967601 PMC6015896

[B81] Wintermans P. C. Bakker P. A. Pieterse C. M. (2016). Natural genetic variation in *Arabidopsis* for responsiveness to plant growth-promoting rhizobacteria. *Plant Mol. Biol.* 90 623–634. 10.1007/s11103-016-0442-2 26830772 PMC4819784

[B82] Xiao J. Luo Y. Xu J. Xie S. Xu J. (2011). *Modestobacter marinus* sp. nov., a psychrotolerant actinobacterium from deep-sea sediment, and emended description of the genus Modestobacter. Int. J. Syst. Evol. Microbiol. 61 1710–1714. 10.1099/ijs.0.023085-0 20802061

[B83] Xing K. Liu W. Zhang Y. J. Bian G. K. Zhang W. D. Tamura T. (2013). *Amycolatopsis jiangsuensis* sp. nov., a novel endophytic actinomycete isolated from a coastal plant in Jiangsu. China. *Antonie van Leeuwenhoek* 103 433–439. 10.1007/s10482-012-9823-y 23053697

[B84] Xing K. Qin S. Bian G. K. Zhang Y. J. Zhang W. D. Dai C. C. (2012). *Pseudonocardia nantongensis* sp. nov., a novel endophytic actinomycete isolated from the coastal halophyte *Tamarix chinensis* Lour. Antonie van Leeuwenhoek 102 659–667. 10.1007/s10482-012-9764-5 22733061

[B85] Xing K. Qin S. Zhang W. D. Cao C. L. Ruan J. S. Huang Y. (2014). *Glycomyces phytohabitans* sp. nov., a novel endophytic actinomycete isolated from the coastal halophyte in Jiangsu. East China. *J. Antibiot.* 67 559–563. 10.1038/ja.2014.40 24736858

[B86] Yamamoto K. Shiwa Y. Ishige T. Sakamoto H. Tanaka K. Uchino M. (2018). Bacterial diversity associated with the rhizosphere and endosphere of two halophytes: *Glaux maritima* and *Salicornia europaea*. *Front. Microbiol.* 9:2878. 10.3389/fmicb.2018.02878 30555434 PMC6282094

[B87] Ye X. Anjum K. Song T. Wang W. Yu S. Huang H. (2016). A new curvularin glycoside and its cytotoxic and antibacterial analogues from marine actinomycete *Pseudonocardia* sp. HS7. *Nat. Prod. Res.* 30 1156–1161. 10.1080/14786419.2015.1047775 26119337

[B88] Zeng J. Xu T. Cao L. Tong C. Zhang X. Luo D. (2018). The role of iron competition in the antagonistic action of the rice endophyte *Streptomyces sporocinereus* OsiSh-2 against the pathogen *Magnaporthe oryzae*. *Microb. Ecol.* 76 1021–1029. 10.1007/s00248-018-1189-x 29679119

[B89] Zhang Y. J. Zhang W. D. Qin S. Bian G. K. Xing K. Li Y. F. (2013). *Saccharopolyspora dendranthemae* sp. nov. a halotolerant endophytic actinomycete isolated from a coastal salt marsh plant in Jiangsu. China. *Antonie van Leeuwenhoek* 103 1369–1376. 10.1007/s10482-013-9917-1 23559043

[B90] Zhao G. Z. Li J. Zhu W. Y. Wei D. Q. Zhang J. L. Xu L. H. (2012). *Pseudonocardia xishanensis* sp. nov., an endophytic actinomycete isolated from the roots of Artemisia annua L. Int. J. Syst. Evol. Microbiol. 62 2395–2399. 10.1099/ijs.0.037028-0 22140165

[B91] Zhao K. Li J. Zhang X. Chen Q. Liu M. Ao X. (2018). Actinobacteria associated with *Glycyrrhiza inflata* Bat. are diverse and have plant growth promoting and antimicrobial activity. *Sci. Rep.* 8:13661. 10.1038/s41598-018-32097-8 30209357 PMC6135863

