# Supplementary material for: Community Composition and Metabolic Potential of Endophytic Actinobacteria From Coastal Salt Marsh Plants in Jiangsu, China
Source: Front Microbiol. 2019 May 14;10:1063. doi: 10.3389/fmicb.2019.01063 (PMC6527748; doi:10.3389/fmicb.2019.01063)

Supplementary Material

Community composition and metabolic potential of endophytic actinobacteria from coastal salt marsh plants in Jiangsu, China

Pan Chen†, Chunmei Zhang†, Xiuyun Ju, Youwei Xiong, Ke Xing, Sheng Qin*

The Key Laboratory of Biotechnology for Medicinal Plant of Jiangsu Province, School of Life Science, Jiangsu Normal University, Xuzhou, Jiangsu, 221116, P. R. China.

† These authors contributed equally to this work

*To whom correspondence should be addressed:

Dr. Sheng Qin

The Key Laboratory of Biotechnology for Medicinal Plant of Jiangsu Province,

School of Life Science,

Jiangsu Normal University

Tel: +86 (0516) 83403515

E-mail: shengqin@jsnu.edu.cn

Table S1. Antifungal activity and biosynthetic genes checking of obtained representative actinobacteria.

Strains	Genus	Activity (mm) against [a]								Presence of genes [b]			
		1[c]	2	3	4	5	6	7	8	PKS I	PKS II	NRPS	Halo
KLBMP 1262	*Amycolatopsis*	-	-	-	-	-	-	-	-	-	-	-	+
KLBMP 1272	*Pseudonocardia*	+++		+	-	++	+	++	++	-	-	-	-
KLBMP 1273	*Pseudonocardia*	-	-	-	+	-	-	-	+	-	-	-	-
KLBMP 1276	*Pseudonocardia*	-	-	-	+	+		++	+	-	-	+	-
KLBMP 1282	*Pseudonocardia*	-	+	-	-	-	-	-	-	-	-	-	-
KLBMP 1283	*Pseudonocardia*	-	+	-	-	-	-	-	-	-	-	-	-
KLBMP 1285	*Pseudonocardia*	+	-	-	+	+	-	+	+	-	-	-	+
KLBMP 1413	*Pseudonocardia*	+	-	-	+	++	+	+	+	-	-	-	-
KLBMP 1325	*Saccharopolyspora*	-	-	-	-	-	-	-	-	-	-	+	-
KLBMP 1478	*Saccharopolyspora*	-	-	-	-	-	-	+	-	+	-	-	-
KLBMP 1333	*Saccharopolyspora*	-	-	-	-	+	-	-	-	+	-	+	-
KLBMP 1337	*Saccharopolyspora*	+	-	-	-	-	-	-	-	-	-	-	-
KLBMP 1287	*Saccharopolyspora*	+	-	-	++	++	+	+++	+	+	-	-	-
KLBMP 1356	*Tamaricihabitans*	-	-	-	-	-	-	-	-	-	-	+	-
KLBMP 1278	*Glycomyces*	-	-	-	-	-	-	-	-	-	-	-	+
KLBMP 1279	*Modestobacter*	-	-	-	-	-	-	-	-	-	+	-	-
KLBMP 1260	*Janibacter*	-	-	-	-	+	-	-	-	-	-	-	-
KLBMP 1288	*Micrococcus*	-	-	-	-	-	-	-	-	-	+	-	-
KLBMP 1309	*Micrococcus*	-	-	-	-	-	-	-	-	-	-	-	+
KLBMP 1290	*Rhodococcus*	-	-	-	-	+	-	-	-	-	-	+	-
KLBMP 1293	*Rhodococcus*	-	-	-	-	-	-	-	-	-	-	+	-
KLBMP 1274	*Kineococcus*	-	-	-	-	-	-	-	-	-	+	-	-
KLBMP 1310	*Gordonia*	-	-	-	-	-	-	-	-	-	-	+	-
KLBMP 1316	*Gordonia*	-	-	-	-	-	-	-	-	-	-	+	-
KLBMP 1292	*Nesterenkonia*	-	-	-	-	-	-	-	-	-	-	+	-
KLBMP 1294	*Prauserella*	-	-	-	-	+	-	-	-	-	-	-	-
KLBMP 1301	*Kocuria*	-	-	-	-	-	-	-	-	-	-	-	+
KLBMP 1304	*Citriococcus*	-	-	-	-	-	-	-	-	-	-	+	+
KLBMP 1311	*Pseudokineococcus*	-	-	-	-	-	-	-	-	-	-	-	+
KLBMP 1314	*Mycobacterium*	-	-	-	-	-	-	-	-	-	-	+	-
KLBMP 1284	*Streptomyces*	-	-	-	-	-	-	-	-	-	-	-	+
KLBMP 1342	*Streptomyces*	-	+	+	+	++	+	++	+	+	-	-	-
KLBMP 1350	*Streptomyces*	+	-	-	-	+	-	+	-	-	-	+	-
KLBMP 1326	*Streptomyces*	+	-	-	++	++		+	-	-	-	-	-
KLBMP 1327	*Streptomyces*	+	++	++	+	+	+		++	-	-	+	-
KLBMP 1334	*Streptomyces*	+	-	-	-	-	-	-	-	-	-	+	-
KLBMP 1328	*Streptomyces*	+++	-	-	+++	++		++	+++	+	-	-	-
KLBMP 1357	*Streptomyces*	+	+	++	+	++	+	+	++	-	+	+	-

KLBMP 1271	*Streptomyces*	-	-	-	-	-	-	-	-	-	-	-	+
KLBMP 1281	*Streptomyces*	-	-	-	-	-	-	-	-	-	-	-	+
KLBMP 1445	*Streptomyces*	+++	-	-	+++	++	+	+++	+++	-	-	-	+
KLBMP 1504	*Streptomyces*	-	-	+	+	+	-	-	+	-	-	-	-
KLBMP 1520	*Streptomyces*	-	-	-	-	-	-	-	-	-	-	+	-
KLBMP 1535	*Streptomyces*	-	+	+	+	+	-	+	+	-	-	-	+
Total		12	6	6	13	18	7	12	13	5	4	16	12

a. Symbols: -, no activity; +, ++, and +++, weak activity, moderate activity, and strong activity, respectively.

b. +, present; -, absent.

c. Eight pathogenic fungal organisms: 1, *Rhizoctonia solani*; 2, *Pyricularia grisea*; 3, *Verticillium dahliae* Kleb; 4, *Lasiodilpodia theobromae* YB3; 5, *Marssonina brunnea* YH1; 6, *Sclerotium* sp. YF2; 7, *Fusarium graminearum*; 8, *Botryospuaeria berengeriana*.

Table S2. Fibrinolytic activity of obtained representative endophytic actinobacteria.

Activity	Strains	Total No.
+++*	KLBMP 1269, KLBMP 1270, KLBMP 1337, KLBMP 1361, KLBMP 1469 KLBMP 1483	6
++	KLBMP 1259, KLBMP 1278, KLBMP 1305, KLBMP 1333, KLBMP 1478, KLBMP 1485, KLBMP 1530, KLBMP 1325, KLBMP 1350, KLBMP 1445, KLBMP 1495	11
+	KLBMP 1262, KLBMP 1267, KLBMP 1272, KLBMP 1301, KLBMP 1327, KLBMP 1336, KLBMP 1341, KLBMP 1342, KLBMP 1354, KLBMP 1356, KLBMP 1414, KLBMP 1471, KLBMP 1472, KLBMP 1474, KLBMP 1535	15
-	Others	47

*：+++：diameter (d) >1.5 cm；++：d=1.0- 1.5 cm；+：d=0-1.0 cm；-：d=0 cm

Fig. S1. Neighbour-joining tree based on almost complete 16S rRNA gene sequences, showing the relationship between strain KLBMP 1440 and its phylogenetic neighbours. Only bootstrap values above 40 %, expressed as percentages of 1,000 replications, are shown at the branch points. Bar 0.005 substitutions per nucleotide position.

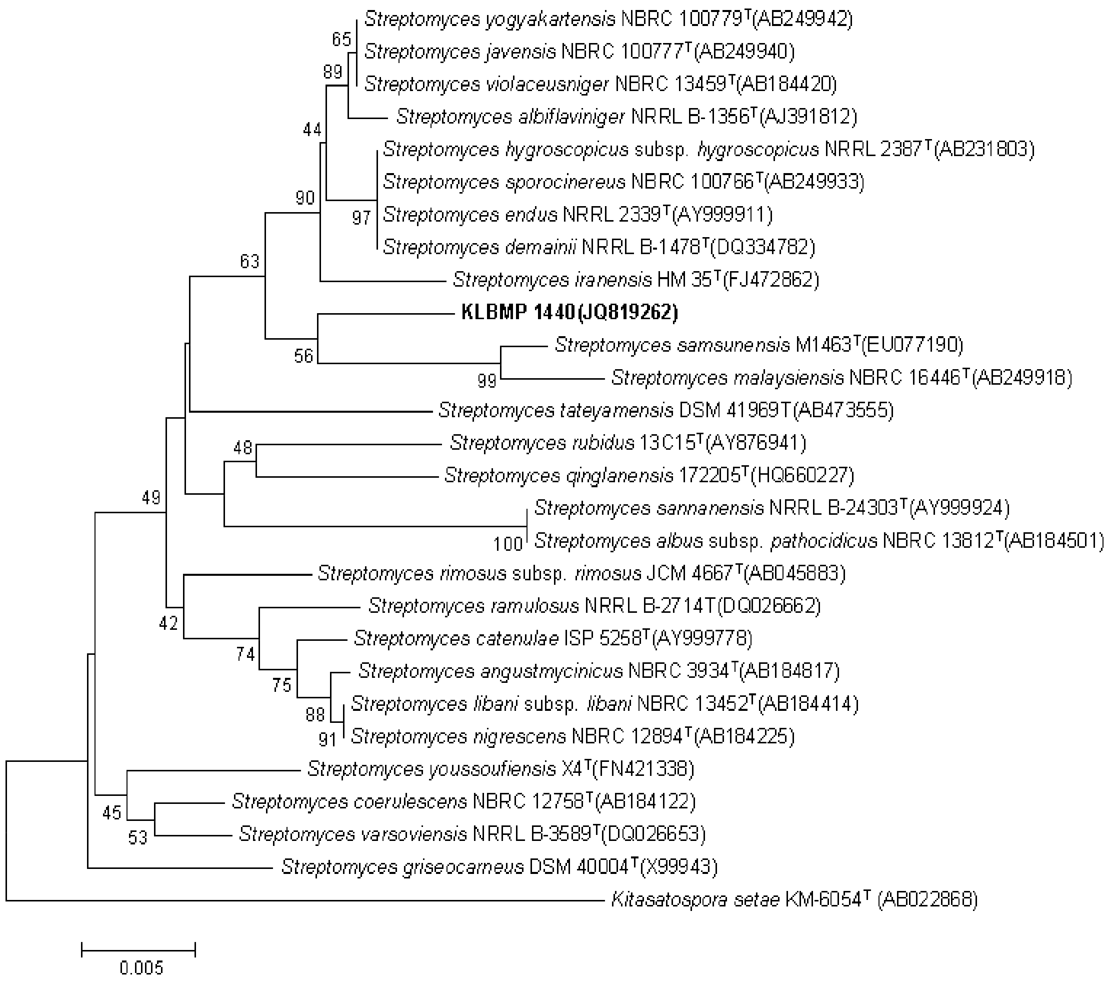

Fig. S2. Neighbour-joining tree based on almost complete 16S rRNA gene sequences, showing the relationship between strain KLBMP 1340 and its phylogenetic neighbours. Only bootstrap values above 40 %, expressed as percentages of 1,000 replications, are shown at the branch points. Bar 0.002 substitutions per nucleotide position.

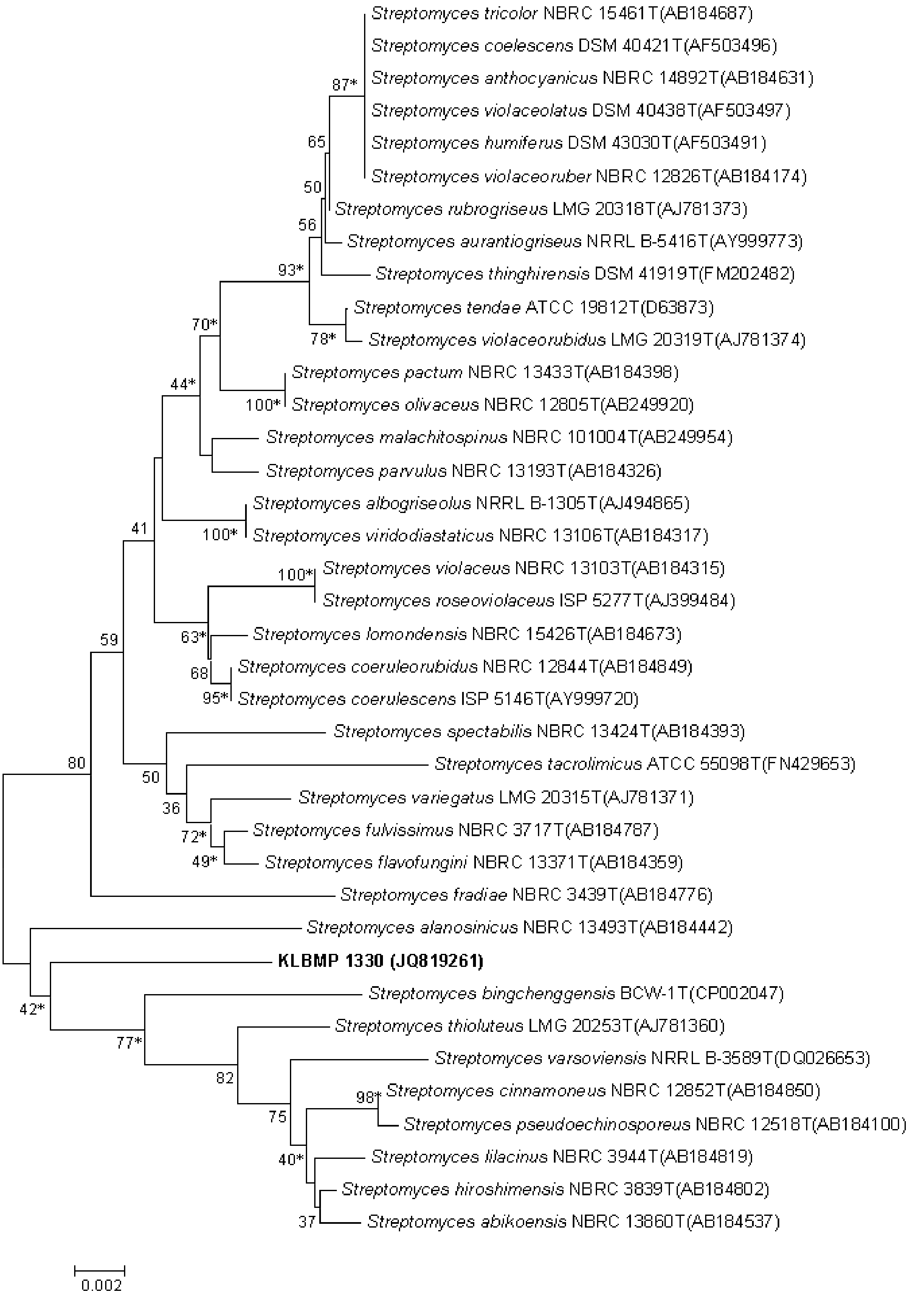

Supplement: Supplementary file 1 [file Data_Sheet_1.pdf]
